# Supplementary material for: Enhanced mechanosensing of cells in synthetic 3D matrix with controlled biophysical dynamics
Source: Nat Commun. 2021 Jun 10;12:3514. doi: 10.1038/s41467-021-23120-0 (PMC8192531; doi:10.1038/s41467-021-23120-0)
Supplement: Supplementary file 1 — Supplementary Information [file 41467_2021_23120_MOESM1_ESM.pdf]

Supplementary Information for

Enhanced mechanosensing of cells in synthetic 3D matrix with controlled biophysical dynamics

Boguang Yang[1,†], Kongchang Wei[1,2,†], Claudia Loebel[3], Kunyu Zhang[1,4], Qian Feng[1,5], Rui Li[1], Siu Hong Dexter Wong[1,6], Xiayi Xu[1], Chunhon Lau[7], Xiaoyu Chen[1,8], Pengchao Zhao[1], Chao Yin[1], Jason A. Burdick[3], Yi Wang[7,], Liming Bian[1,9,10,*]*

[1] Dr. B. G. Yang, Dr. K. C. Wei, Dr. K. Y. Zhang, Dr. Q. Feng, Dr. R. Li, Dr. D. S. H. Wong, Dr. X. Xu, Dr. C. Yin, Dr. P. C. Zhao, Dr. X. Y. Chen, Prof. L. Bian
Department of Biomedical Engineering, The Chinese University of Hong Kong, Shatin, New Territories, Hong Kong, China

[2] Dr. K. C. Wei
Empa, Swiss Federal Laboratories for Materials Science and Technology, Laboratory for Biomimetic Membranes and Textiles, Lerchenfeldstrasse 5, CH-9014, St. Gallen, Switzerland

[3] Dr. C. Loebel, Prof. J. A. Burdick
Department of Bioengineering, University of Pennsylvania, Philadelphia, PA, USA.

[4] Dr. K. Y. Zhang
Department of Materials Science and Engineering, Johns Hopkins University, Baltimore, MD 21218, USA.

[5] Dr. Q. Feng
Key Laboratory of Biorheological Science and Technology, Ministry of Education College of Bioengineering, Chongqing University, Chongqing, 400044, China.

[6] Dr. D. S. H. Wong
Department of Biomedical Engineering, The HongKong Polytechnic University, HongKong, China.

[7] C. H. Lau, Prof. Y. Wang
Department of Physics, The Chinese University of Hong Kong, Shatin, New Territories, Hong Kong, China

[8] Dr. X. Y. Chen
Department of Mechanical Engineering, Massachusetts Institute of Technology, Cambridge, MA, 02139, USA.

[9] Prof. L. Bian
Shenzhen Research Institute, The Chinese University of Hong Kong, Hong Kong, China

[10] Prof. L. Bian
China Orthopedic Regenerative Medicine Group (CORMed), Hangzhou, Zhejiang, China

E-mail: yiwang@cuhk.edu.hk
E-mail: lbian@cuhk.edu.hk

† These authors contributed equally to this work.

Keywords: cell-adaptable hydrogels, 3D cell culture, reversible crosslink binding kinetics, stem cell mechanosensing

Materials. Sodium hyaluronate (NaHA) was purchased from Freda® Biotechnology Co., Ltd. (80 kDa). Dowex 50W, tetrabutylammonium hydroxide (TBAOH), di-tert-butyl dicarbonate (Boc Anhydride), sodium chloride (NaCl), 1-adamantaneacetic acid (ADA), cholic acid (CA), sodium hydroxide (NaOH), β-cyclodextrin (β-CD) and acryloyl chloride were obtained from J&K Scientific. 4-Dimethylaminopyridine (DMAP) was purchased from Aladdin. Dimethyl sulfoxide (DMSO), hydrochloric acid (HCl), and deuterium oxide (D_2O) were acquired from Fisher Scientific. α-minimum essential medium (α-MEM), Dulbecco's phosphate-buffered saline (DPBS), penicillin, streptomycin, L-glutamine, fetal bovine serum (FBS), and calcein AM were obtained from Gibco. Methacrylic anhydride (94%), TRIzol, propidium iodide, tri-Boc-hydrazinoacetic acid and the other chemicals were purchased from Sigma-Aldrich unless otherwise specified. The DAB Substrate Kit for peroxidase and VECTASTAIN ABC kit were purchased from Vector Lab. The human mesenchymal stem cells (hMSCs) were purchased from Lonza.

Synthesis of acrylated β-cyclodextrin (Ac-β-CD). Ac-β-CD was synthesized according to a previously reported method [1]. Briefly, 10 g of β-CD was added to 150 mL of DMF with 7 mL of TEA added into the solution. The mixture was stirred and cooled to 0°C before 5 mL of acryloyl chloride was added into the solution. After stirring for 6 h, the mixture was filtered to remove the trimethylamine hydrochloride, and the clear solution obtained was concentrated to approximately 20 mL by vacuum rotary evaporation. Next, the solution was dripped into 600 mL of acetone to precipitate the modified cyclodextrin. The precipitate was washed several times with acetone and vacuum dried for 3 days. The substitution degree (DS) of the CD (1.2) was confirmed by ^{1}H NMR (Bruker Advance 400 MHz spectrometer).

Synthesis of adamantane modified hyaluronic acid (HA-ADA). HA-ADA was synthesized according to a previously reported method with minor modifications [2, 3]. Sodium hyaluronic acid (HA-Na, 2.0 g) was treated with Dowex resin (6.0 g) for ion exchange in the water phase and then neutralized by tetrabutylammonium hydroxide (TBAOH) to produce the HA-TBA aqueous solution (pH = 7.0), which was soluble in DMSO after lyophilization. HA-TBA (1.0 g, 1.4 mmol disaccharide repeat units, 1 equiv), 1-adamantaneacetic acid (0.82 g, 4.2 mmol, 3 equiv), and 4-dimethylaminopyridine (DMAP, 0.13 g, 1.05 mmol, 0.75 equiv) were dissolved in 100 mL of anhydrous DMSO at room temperature, and di-tert-butyl dicarbonate (BOC_2O) was added slowly under nitrogen protection at room temperature. The mixture was heated to 45°C and maintained for 24 h. After cooling to room temperature, the reaction

mixture was directly dialysed against DMSO, NaCl (*aq*), and then DI water to remove all unreacted small organic compounds. The solution was then frozen and lyophilized to yield the product as a white solid. The degree of modification was quantified by ^{1}H NMR from the integration ratio between the ethyl multiplet of adamantane (δ = 1.50-1.85, 12H) and the HA backbone (δ = 3.20-4.20, 10H) (Supplementary Fig. 1a).

Synthesis of cholic acid modified hyaluronic acid (HA-CA). HA-TBA (1.0 g, 1.4 mmol disaccharide repeat units, 1 equiv), cholic acid (1.72 g, 4.2 mmol, 3 equiv), and 4-dimethylaminopyridine (DMAP, 0.13 g, 1.05 mmol, 0.75 equiv) were dissolved in 100 mL of anhydrous DMSO at room temperature, and di-tert-butyl dicarbonate (BOC_2O) was added slowly under nitrogen protection at room temperature. The mixture was heated to 45°C and maintained for 24 h. After cooling to room temperature, the reaction mixture was directly dialysed against DMSO, NaCl (*aq*), and then DI water to remove all unreacted small organic compounds. The solution was then frozen and lyophilized to yield the product as a white solid. The degree of modification was determined by ^{1}H NMR from the integration ratio between the methyl group of cholic acid (δ = 0.59, 3H) and the HA backbone (δ = 3.20-4.20, 10H) (Supplementary Fig. 1b).

Synthesis of methacrylated HA-ADA and methacrylated HA-CA. Synthesis of methacrylated HA-ADA and methacrylated HA-CA are similar according to a previous report [4]. In short, an excess of methacrylic anhydride was added to a 1% (w/v) HA-ADA or HA-CA solution. Next, the mixture was stirred for approximately 4 h while the pH was kept between 8.0 ~ 8.5 with 2M NaOH adjustment. The product was purified via dialysis against DI water and then freeze dried. The methacrylation degree of the HA-ADA or HA-CA was calculated using ^{1}H NMR (Supplementary Fig. 1c and d).

Synthesis of HA-ADA-cRGD and HA-CA-cRGD. Through Michael Addition reaction, the bioactive thiol-terminated RGD peptides were covalently coupled on the methacrylated HA-ADA or methacrylated HA-CA chain. Next, the remaining methacrylates were blocked by adding β-mercaptoethanol to avoid chemical crosslinking through methacrylates, which would affect the structure of the hydrogel. ^{1}H NMR spectra revealed the HA-ADA-cRGD chemical structures (Supplementary Fig. 1e).

Isothermal Titration Calorimetry (ITC). Titrations were performed on MicroCal iTC200 isothermal titration calorimeter (ITC) at room temperatures (25 °C). Solutions of ac-β-CD in PBS were titrated into solutions of HA-ADA (or HA-CA) in PBS. The syringe needle was equipped with a flattened, twisted paddle at the tip, which ensured continuous mixing of the solutions in the cell rotating at 1000 rpm. Titrations were carried out by consecutive 2 μL injections of an aqueous ac-CD solution from the syringe into the sample cell containing an aqueous HA-ADA (or HA-CA) solution. A total of 20 consecutive injections were performed. The delay between two consecutive injections was 150 s. The concentration changes due to injections have been taken into account when analyzing experimental data by the NanoAnalyze software.

Preparation of MeHA hydrogels. For the static MeHA hydrogel, MeHA were first dissolved in PBS (pH = 7.4) and then mixed with the photoinitiator I2959 (final concentration: 0.05 wt%). The precursor was loaded in moulds (13 mm in diameter). After 11 min of UV irradiation (7 mW/cm^2), disc-like MeHA hydrogels were obtained.

Swelling ratios of the hydrogels. Swelling ratios of the different dynamic hydrogels were measured by immersing the hydrogels in DI water for 48 hours at room temperature, and then the samples were frozen and lyophilized for 48 h. The swollen ratio was calculated by the following formula:

$$\text{Swelling ratio} = (W_{\text{wet}} - W_{\text{dry}})/W_{\text{dry}} \times 100\% \qquad (1)$$

where W_{wet} and W_{dry} represent the weights of the swollen hydrogels and dried hydrogels, respectively.

Mechanical characterization. Rheological characterizations were performed using a Kinexus Rheometer from Malvern. Samples were formed in situ by pipetting a 30 µL monomer solution between the bottom Kinexus Rheometer plate and an 8 mm flat plate, and the experiment was initiated after photopolymerization. Evolution experiments were performed at 25°C. Frequency sweeps were performed at 1% strain.

The stress relaxation properties of the samples were measured from compression tests of the gel discs (8 mm in diameter, 2 mm thick). The gel discs were compressed to 15% strain with a deformation rate of 0.3 mm s^{-1}.

Molecular dynamics (MD) modelling. Our simulation box of the HA-ADA hydrogels contained six CD-ADA pairs, with the guest molecules evenly distributed over three HA segments, each segment consisting of seven disaccharide units. The resulting system matches the density (4% w/v) and guest DS (30%) of HA-ADA used in experiments. More details of this all-atom model with explicit water are provided at the end of this section. With all CD-ADA pairs in the bound state and the hosts un-oligomerized, we first launched four replicas of 250-ns simulations to determine the distribution of host molecules within the hydrogel, in order to identify a representative number n in the n-host complex oligomerized via acrylates. With the aggregated 1-µs trajectories, DBSCAN clustering analysis based on the minimum pairwise distance between the hosts show that clusters with $n \geq 4$ were rarely formed (probability < 2%), reflecting the steric clash between adjacent host molecules. Here, we chose $n = 3$ in the following MD simulations, while both the $n = 2$ and $n = 3$ cases were investigated in the KMC calculations.

Three replicate simulations of ~700 ns to 1 µs were then performed for the HA-ADA system, where the six host molecules formed two complexes, each containing $n = 3$ hosts oligomerized via acrylates. With the host and guest molecules initially in the unbound state, these simulations were designed to examine their spontaneous binding and to provide starting structures for the subsequent pulling simulations. During these simulations, over 80% of the initially free CD and ADA molecules were found to spontaneously bind to each other within hundreds of nanoseconds. This timescale is consistent with the k_{on} value of 10^8 $M^{-1}s^{-1}$ of CD-ADA binding. Initiating from snapshots obtained from these simulations, we then applied an external force ranging from 10 to 102 pN on HA segments with either free (unbound) or bound host–guest pairs. These short (5-ns) pulling simulations were designed to explore the relationship between applied force and the dynamics of CD-ADA complexation. Through altogether 480 such simulations, we found that even tens of piconewton forces were sufficient to induce directed movement of those HA segments with unbound host and guest molecules (**Supplementary Table 3**). This result indicates that for HA segments with unbound host-guest crosslinks, cells needed to only apply small ($\ll$ 100 pN) forces to

move them "out of the way". In clear contrast, the same forces mentioned above generally were not strong enough to break bound CD-ADA pairs in our simulations. Out of sixty 5-ns runs during which a force of 102 pN was applied along a given HA segment, host–guest unbinding was only observed in two simulations, while forces ranging from 10 to 50 pN were unable to break any bound host–guest pair (**Supplementary Table 3**). This result is in qualitative agreement with a straightforward estimation using the Bell model [5, 6]. Specifically, we consider

$$k_{off}=k_{off,0}\exp(F/F_0) \qquad (2)$$

where k_{off} is the dissociation rate constant between a host–guest pair under an external force F, while $k_{off,0}$ is the base k_{off} measured under zero external force. F_0 represents the rupture force between the given host-guest pair. With F_0 = 102 pN for CD-ADA [7], $k_{off,0}$ = 10^3 s^{-1}, and F ranging from 10 to 102 pN, the resulting k_{off} ranges from 1.1×10^3 s^{-1} to 2.7×10^3 s^{-1}, corresponding to a characteristic time of 0.9 ms to 0.4 ms for a single bound CD–ADA pair to become free.

As mentioned earlier, sixty 5-ns simulations were performed for each value of the applied force (10, 20, 50, and 102 pN) with a selected CD-ADA pair in the unbound state, resulting in a total of 240 pulling simulations. Correspondingly, another set of 240 (sixty at each of the above forces) 5-ns pulling simulations were performed with all CD-ADA pairs in the bound state. During all of the above simulations, a force with a constant magnitude was applied along one end of a given HA segment. The direction of the force was approximately tangential to the HA chain, *e.g.*, along the vector pointing from the C4 atom of the second 2-acetyl-2-deoxy-β-D-glucosamine to the C4 atom of the first β-D-glucuronic acid in the given HA chain (Fig. 3a and Supplementary Fig. 9a). The central carbon atom of each 3-host complex was constrained with a force constant of 1.0 kcal/mol/Å^2 to avoid the overall translation of the system. The correlation coefficient between the force vector and the displacement vector of the guest molecule closest to the HA end being pulled was calculated as the dot product of the two unit vectors and was averaged over a short window (1 ns) (**Supplementary Table 3**). To estimate the "speed" of chain movement, we calculated the diffusion coefficient D of the above guest molecule through a linear regression model fit to its mean square displacement ($< r^2 >$) according to $< r^2 >= 2bDt$, where $b = 3$ stands for the dimension. Finally, the change in distance between the aforementioned guest molecule and its initially nearest unpaired host was measured as the following: at the beginning of each pulling simulation, we determined the distance between the six host molecules and the single free guest molecule closest to the HA end being pulled; the distance between that guest and its initially nearest unpaired host was determined again in the last 0.5 ns of the simulation. The difference between these two measurements, averaged over all replicate simulations, were then reported (Supplementary Fig. 9b). This distance calculation was repeated for sixty 5-ns equilibrium simulations initiated from the same structures as the pulling simulations, only without any applied force. These control simulations produced the F = 0 data point in Supplementary Fig. 9b.

Apart from the HA-ADA system, MD simulations were also performed on the HA-CA system, where cholic acid, instead of adamantane, served as the guest molecule. Before launching the HA-CA simulations, however, we first determined the potential of mean force (PMF), *i.e.*, the free energy landscape of a single CA molecule binding to β-CD. This was achieved through 9.4-μs adaptive biasing force (ABF) [8, 9] calculations, with the reaction coordinate chosen as the *z*-axis projection of the center-of-mass distance vector between the steroid body of CA and β-CD (the ring of β-CD was held in the *xy*-

plane, see Supplementary Fig. 8). Since the host molecules were acrylated on the narrow ends in our hydrogel, the guest entrance faces less steric hindrance through their wide ends. This is confirmed by the aforementioned spontaneous binding simulations of CD-ADA, where all guest molecules were found to enter from the wide end of β-CD. The same orientation was thus adopted for CA-CD to mimic the condition in our hydrogel. As shown in Supplementary Fig. 8, our ABF calculations locate an overall binding energy minimum when the CA molecule's five-membered ring D and hydrophobic tail is inside the host cavity, in agreement with its inserted portion revealed by NMR ROESY spectra [10, 11]. The computed binding free energy at this location (-6 kcal/mol) is in good agreement with the binding free energy corresponding to K_{eq} of CD-CA measured in our hydrogel from ITC experiment (-6.6 kcal/mol, see Supplementary Fig. 2 and Supplementary Table 5).

Upon confirming the binding mode of CD-CA, we constructed the HA-CA system by replacing the guest molecules in the HA-ADA system with CA. Similar to HA-ADA pulling simulations, an external force was applied on HA segments with either free (unbound) or bound CD-CA pairs, in order to examine the relation between the applied force and dynamics of the host-guest complexation. Specifically, 240 5-ns pulling simulations were performed with forces of 10, 20, 50 and 102 pN applied on HA segments with free CD-CA, following the same setup as the HA-ADA simulations. However, only the largest force (102 pN) was applied in the corresponding simulations with bound CD-CA — since the CD-CA pair has smaller k_{off} than CD-ADA, unbinding is not expected to occur at lower forces. Indeed, no unbinding event of the CD-CA complexation is recorded even in the sixty 5-ns pulling simulations performed at F = 102 pN (**Supplementary Table 3**).

In all MD simulations, the CHARMM36 carbohydrate [12, 13] and general [14] force fields were employed to model the HA-ADA and HA-CA systems. Specifically, β-cyclodextrin was created by linking seven α-D-glucose via the 14ab patch in CHARMM36, with its initial structure obtained from the protein data bank (PDB code 3gct). The HA chain was created by linking β-D-glucuronic acid and 2-acetyl-2-deoxy-β-D-glucosamine via the 13bb and 14bb patches alternatively. The acrylate, adamantane and cholic acid parts were all parameterised via the CGenFF website [14-16], which produced small penalty values up to 10 to 12. A small number of remaining missing parameters were obtained via analogy. All simulations were performed with the 2.12 or 2.13 release of NAMD [17]. A time step of 2 fs was adopted in all simulations with bonds involving hydrogen atoms constrained using RATTLE [18] and water geometries maintained using SETTLE [19]. The multiple-time-stepping algorithm was used with short-range forces calculated every step and long-range electrostatics calculated every two steps. The cutoff for short-range nonbonded interactions was set to 12 Å, with a switching distance of 10 Å. Assuming periodic boundary conditions, the Particle Mesh Ewald (PME) method [20] with a grid density of at least 1/Å^3 was employed for computation of long-range electrostatic forces. Langevin dynamics with a damping coefficient of 1 ps^{-1} was used to keep the temperature constant at 300 K, while a Nosé-Hoover-Langevin piston [21] was used to maintain the pressure at 1 atm. During ABF calculations, the z coordinate of atom O4 in each unit of β-CD was constrained with a spring constant of 1.0 kcal/mol/Å^2. The 28.6-Å reaction coordinate (from z = -11 Å to z = 18.6 Å) was divided into nine windows, with an overlap of at least 1.0 Å between adjacent windows. ABF calculations ranging from 600 ns to 1.8 μs were performed for each window, resulting in a total of 9.4 μs sampling. Gradients were

collected with a full Samples number of 2000 and a bin width of 0.2 Å. The resulting PMF has an asymmetry of ~3 kcal/mol between its two ends (thin gray curve in Supplementary Fig. 8), reflecting the error in the calculation. Assuming that more errors occur in regions with larger gradients, we obtained a symmetrized PMF by distributing the ~3 kcal/mol error according to the magnitude of the gradients along the reaction coordinate (thick black curve in Supplementary Fig. 8). Considering this error in PMF, we examined a two-orders-of-magnitude range of k_{off} in our subsequent KMC calculations (Supplementary Fig. 9h). Finally, we note that in order to avoid the 'wandering ligand' issue [22], the top and bottom carbon atoms (C3 and CD) of the CA molecule were restrained in a cylinder in the three outermost windows at each end of the PMF. Placed at the center of β-CD, this cylinder has a diameter of 8 Å, which restricts both the location and orientation of CA. The corresponding adjustment for the loss in translational and rotational entropy sets the two ends of the PMF at ~1.7 kcal/mol, with the standard state in bulk solution taken as reference (0 kcal/mol).

Kinetic Monte Carlo (KMC) calculation. Typical filopodium contains a bundle of 15 to 20 actin filaments packed hexagonally and crosslinked by proteins such as fascin, with the distance between adjacent actin filaments estimated at 7-9 nm [23-27]. Here, we considered an actin bundle with 15-20 filaments spaced 8 nm apart. Assuming that n host molecules are oligomerized together via an acrylate chain, these n-host complexes form a set of 3D grids that crosslink the guest-decorated HA chains. Through an exhaustive search we estimated the number of such n-host complexes a single actin filament might encounter. With n = 2, a single actin filament faces 1, 2, 3 and 4 such complexes at a probability of 0.05, 0.54, 0.28 and 0.13, respectively. With n = 3, the corresponding probability is 0.23, 0.66, 0.10, and 0.01, respectively. Individual KMC calculations were performed for each of the four cases corresponding to a given n, and the resulting numbers of 'fast-enough' gate-opening events (Fig. 3f) were combined using the aforementioned probabilities as weights (Supplementary Fig. 9g). As hydrogels studied experimentally are likely mixtures of n-host complexes, their behaviours can be expected to lie within the range of the KMC results obtained here. It should be noted that out of each n-host complex, only n-1 host-guest pairs need to unbind in order to "free" the corresponding grid. Therefore, with four 2-host complexes, 4 host–guest pairs (1 from each 2-host complex) should unbind; while with four 3-host complexes, 8 host–guest pairs (2 from each 3-host complex) should unbind. These up to 8 host–guest pairs thus represent an undividable, minimum set, *i.e.*, "a gate" that must open for individual actin polymerization to occur. During such polymerization, G-actin monomers are added onto the barbed end of Filamentous (F)-actin. Each newly added G-actin extends the F-actin filament by approximately 2.7 nm [28], which is well below the average spacing (~5 nm) between the n-host complexes in our hydrogels. Therefore, a single actin polymerization event requires only one gate to open. Notably, all actin filaments within the same filopodium need not to be perfectly synchronized in their growth—as long as the gates open fast enough for each individual actin polymerization event, protrusion of the entire filopodium may continue.

Next, we estimated the maximum force cells could exert on a single HA chain. Such force is ultimately transmitted via integrin receptors, which bind to ligands in the surrounding microenvironment. In our hydrogels, RGD peptides decorating the HA chains serve as the ligands for integrin binding. Given a typical molecular weight of 80 kDa for a single HA chain and a DS value of 3% for the RGD peptides, we estimated that there were on average six RGD on a single HA chain. The average spacing between

these peptides is over 30 nm, which should be more than sufficient to pack integrin receptors, given that the latter has diameters of 10 nm [29] and that they have been shown to pack less than 20 nm apart in platelets [30]. Therefore, we estimated that there were up to six integrin–RGD bonds on a single HA chain. The force transmitted by individual integrin receptors has been found to be 110 ± 9 pN in rat embryonic fibroblasts [31], although smaller values, ranging from 40 pN [32] to only 2-4 pN [33] have also been reported. A recent study attempting to resolve this discrepancy has found that not all integrin-ligand bonds bear the same load: while the vast majority are under small tension of 1-7 pN, approximately 20% bear greater tension [34]. In our case, it is likely that cells "pull the hardest" only on those few HA chains immediately blocking its spreading, whereas once host–guest crosslinks on a given HA segment are broken, a much smaller force can be applied to displace that segment, as suggested by the results of our MD simulations. In summary, we estimated that the forces exerted by cells on a single HA chain ranged from tens of piconewtons up to about 660 pN. Due to load sharing, the force felt by an individual host–guest pair is inevitably smaller than the above values. Nonetheless, load sharing can only be achieved when an initially loose HA segment becomes stretched (Supplementary Fig. 9a). As a result, it is likely that only a small number of host–guest pairs share the load at a time, which then unbind to shift it to the next batch of host–guest pairs. As we could not determine the exact number of load-bearing host–guest pairs, we considered the entire range of forces mentioned above, bearing in mind that the maximum of 660 pN is an overestimation. As can be seen from the comparison of Fig. 3f and Supplementary Fig. 9e-h, while the distribution of the gate-opening time has a strong dependence on the applied force, the probability of achieving 'fast-enough' gate-opening events in our hydrogels shows a much weaker dependence. This later result may have arisen from the relationship between the actin polymerization timescale and kinetic constants of the two types of host-guest complexation studied here — the CD-ADA pairs are nearly always 'fast enough', whereas the CD-CA pairs, under the above range of forces, are generally not. This renders the probability for gate-opening to occur within the estimated actin polymerization timescale dominated by the presence of the former host-guest pairs.

As a final step before launching the KMC calculations, we estimated the kinetic constants of CD-CA. While these constants have been reported for a variety of bile acids [11, 35], they all corresponded to the association and dissociation from the 'thin' carboxylate side of the molecules. As the carboxylate group is covalently attached to the HA chains in our hydrogel, association and dissociation had to occur through the bulkier steroid body of CA. According to our ABF calculations (Supplementary Fig. 8), the free energy barrier (E_b) against such dissociation is estimated to be ~21 kcal/mol, about 8 kcal/mol higher than the energy barrier against dissociation from the carboxylate side, the latter of which was estimated to be ~13 kcal/mol [11]. Assuming that k_{off} is proportional to $\exp(-E_b/k_BT)$, where k_B is the Boltzmann's constant and T is the temperature, we estimate that k_{off} for dissociation from the steroid body side of CA is ~1.3×10^{-3} s^{-1}, which is about 10^6 times slower than that reported by Schönbeck for dissociation from the carboxylate side [11]. Given the error in our PMF, we also examined k_{off} an order of magnitude larger or smaller than this estimated value, *i.e.*, 1.3×10^{-4} s^{-1} and 1.3×10^{-2} s^{-1}, in our KMC calculations (Supplementary Fig. 9h). The corresponding k_{on} values were obtained by multiplying k_{off} with the equilibrium binding constant of the CD-CA pair (K_{eq} = 2.93×10^4 M^{-1}) converted from their binding free energy found in our ABF calculations. The rupture force between CD and CA was estimated to be

84.8 pN according to the value of K_{eq} and the cylindrical integration approach given in Auletta et al [7].

Based on the parameters described above, we carried out a series of kinetic Monte Carlo calculations to determine the gate-opening time, *i.e.*, the time it takes for the required number of host-guest pairs to be all in the unbound state. The effect of external force on k_{off} was again described using the Bell model [5, 6]. All KMC calculations were initiated with host-guest pairs either in the bound or unbound state according to the probability determined by their equilibrium binding constants. In mixed hydrogels, a given host–guest pair is assigned randomly to be either CD–ADA or CD–CA according to their mixing ratio. With force-induced diffusion of unbound guests considered in the next section, each host–guest pair was allowed to independently switch between bound and unbound states. The time it takes for such switches is determined by k_{on} and k_{off} according to the standard KMC algorithm [36]. More specifically, the "survival" probability that a host–guest pair formed at t = 0 remains bound at time t is given by [36]

$$p_b(t) = \exp(-k_{off}t) \qquad (3)$$

Correspondingly, the probability distribution function for the time of "first escape", *i.e.*, when the host–guest pair first breaks up, is

$$p_{b2u}(t) = k_{off} \exp(-k_{off}t) \qquad (4)$$

where the subscript b2u stands for the switch from the bound to unbound state. In a KMC calculation, the above probability distribution function is sampled by drawing a random number r on the interval (0,1) and then let $t = -(1/k_{off})\ln(r)$. Similarly, the probability distribution function for the time to switch from the unbound to bound state is

$$p_{u2b}(t) = k_{on}[H]\exp(-k_{on}[H]t) \qquad (5)$$

where $[H]$ is the free host concentration. Here, since host molecules are much more mobile than the guests (the latter are covalently bonded to HA chains while the former are not) and given that cells could only exert forces on a small number of HA chains, we assume that the overall equilibrium of host-guest binding within the hydrogel is maintained, which allow us to determine the free host concentration based on K_{eq} and the total host/guest concentration. In the case of mixed HA-ADA and HA-CA hydrogel, we assume that CD–ADA and CD–CA binding is each in equilibrium, and the resulting free host concentration is obtained by solving a cubic equation involving the mixing ratio and equilibrium binding constants of both types of host–guest pairs.

When a given guest is in the unbound state, its probability of being moved "out of the way" by the applied external force is estimated using the force-dependent diffusion coefficient obtained from our MD simulations. More specifically, we consider the 3D distribution probability of a particle diffusing from the origin as [37]

$$P(r,t) = \frac{r^2}{(4\pi)^{\frac{1}{2}}(Dt)^{\frac{3}{2}}} e^{-\frac{r^2}{4Dt}} \qquad (6)$$

Integrating from $r = 0$ to $r = R$ yields the "survival" probability that the particle remains within a radius R at a given time t:

$$P_{survival} = \mathrm{erf}\left(\frac{R}{\sqrt{4Dt}}\right) - \frac{R}{\sqrt{\pi Dt}} e^{-\frac{R^2}{4Dt}} \qquad (7)$$

With $R = 8$ nm corresponding to the typical actin filament spacing and the force-dependent diffusion coefficient D obtained from our MD simulations, $P_{survival}$ is fit to an exponential form to produce an "effective diffusion rate constant" k_D. While it is clearly an approximation, the fitted curve nonetheless agrees well with the original

$P_{survival}$ (Supplementary Fig. 9c). Based on such fitting performed on MD results (F = 10, 20, 50, 102 pN) of HA-ADA and HA-CA, a linear F vs. k_D relationship (Supplementary Fig. 9d) is observed and subsequently used to estimate k_D at a given applied force. For mixed hydrogels, a weighted average of k_D values of HA-ADA and HA-CA were used based on their given mixing ratio. k_D is set to zero at $F = 0$ and no negative k_D is invoked. Thus, for an unbound guest, the next event (binding versus force-directed diffusion) to occur is chosen based on the standard KMC algorithm, *i.e.*, two random time drawn according to $t_1 = -(1/k_{on}[H])\ln(r_1)$ and $t_2 = -(1/k_D)\ln(r_2)$, where r_1 and r_2 are two random numbers in the range (0, 1), is compared and the event with a shorter time is chosen. It is worth pointing out that the values of k_D are generally orders of magnitude larger than k_{off} of the host-guest crosslinks. This "separation of timescale" allows the approximation of neglecting the "in-coming" flux of guests, since the latter is largely determined by the rate at which unbound guests become available, *i.e.*, k_{off}.

Based on the above equations, 100,000 KMC runs are performed to determine the distribution of the first time that the required number of host–guest pairs are all in the unbound state, *i.e.*, the time it takes for a gate-opening event to first occur. Specifically, with M n-host complexes, the required number of host-guest pairs correspond to at least n-1 out of the n host–guest pairs within each of the M complexes. If a gate-opening event is not detected in a given KMC run, the sampling time of that run is extended to at least ~0.1 s, *e.g.*, ten times the typical timescale of actin polymerization. The probability of achieving gate-opening events faster than ~0.01 s, *i.e.*, faster than actin polymerization, is then collected from the total of 100,000 KMC runs. These calculations are performed individually for each of the four cases of M (M = 1, 2, 3, or 4) corresponding to a given n (n = 2 or n = 3). The final probabilities of 'fast-enough' gate-opening are then obtained from their weighted averages as described earlier.

Western blotting. Total protein was extracted using RIPA Lysis and Extraction Buffer (Thermo Fisher Scientific) and Halt Phosphatase Inhibitor Cocktail (Thermo Fisher Scientific) on ice for at least 30 min according to the manufacturer's protocol. Later, the supernatant was collected after centrifugation at 12,000 g and 4°C, and the protein concentration was measured using a Pierce BCA Protein Assay Kit (Thermo Fisher Scientific). Next, all the samples were electrophoresed through a 10% Bis-Tris polyacrylamide gel before being transferred to a 0.45-mm PVDF membrane (Millipore, Billerica, Massachusetts, USA). After three washes with PBS supplemented with Tween-20, the PVDF membranes were blocked with a 5% fat-free milk powder solution for 2 h at room temperature and then incubated with various primary antibodies overnight at 4°C with gentle rocking. Horseradish peroxidase-coupled secondary antibody (anti-mouse secondary antibody, 1:800, Santa Cruz, Dallas, Texas, USA) was then applied and incubated with membranes for 120 min at room temperature with gentle rocking. Fluorescence signals were displayed with an enhanced chemiluminescence (ECL) substrate (Millipore) under an imaging system (Bio-Rad, Hercules, CA, USA). Densitometric analysis was conducted using open source ImageJ software (NIH). The primary antibodies used include Runx 2 (1:600; Santa Cruz Biotechnology, cat. # sc-101145), ALP (1:600; Santa Cruz Biotechnology, cat. # sc-271431) and GAPDH (1:600; Santa Cruz Biotechnology, cat. # sc-32251).

Gene expression analysis. For gene expression analysis, the samples were harvested and homogenized in TRIzol reagent, and RNA was extracted according to the manufacturer's instructions. The RNA concentration was then determined using a ND-

1000 spectrophotometer (Nanodrop Technologies). One hundred ng of RNA from each sample was reverse transcribed into cDNA using a RevertAid First Strand cDNA Synthesis Kit (Thermo). Real-time PCR was performed on an Applied Biosystems 7300 Real-Time PCR system using Taqman primers and probes specific for GAPDH (housekeeping gene), vinculin and the osteogenic marker genes (Runx 2, Col I and Runx 2). The information of the primers is listed in the **Supplementary Table 6**. The relative gene expression was calculated using the ΔΔCT method, where fold differences were calculated using the expression of $2^{-\Delta\Delta CT}$.

Supplementary Table 1. Hydrogel compositions used in the study.

Group name	HA conc. (%)	Crosslinking density (%)	Storage modulus (kPa)
MeHA-cRGD	4	~30	~9
HA-ADA-cRGD	4	~30	~1
HA-CA-cRGD	4	~30	~1

Conc.: Concentration

Supplementary Table 2. Western blot analysis of vinculin and β_1 integrin protein expression in hMSCs after 5 days of osteogenic culture in hydrogels prepared with different lifetime crosslinks.

	Day 1	Day 3	Day 5
**Relative intensity of Vinculin (HA-ADA-cRGD)**	0.94	1.30	1.30
**Relative intensity of Vinculin (HA-CA-cRGD)**	1.10	0.95	0.82
**Relative intensity of Integrin β_1 (HA-ADA-cRGD)**	0.82	0.95	1.35
**Relative intensity of Integrin β_1 (HA-CA-cRGD)**	0.38	0.53	0.65

Supplementary Table 3. MD simulation results showing the effect of applied force on a single host-guest pair. For a given value of applied force, sixty 5-ns simulations were performed on the HA-ADA system with all CD-ADA pairs initially in the bound state. Among these 240 simulations, unbinding events were only observed in two simulations with F = 102 pN. Sixty 5-ns simulations with F = 102 pN on the HA-CA system with all CD-CA pairs initially in the bound state revealed no unbinding event. Simulations with lower forces were not performed for the HA-CA system with bound CD-CA pairs. For both HA-ADA and HA-CA, a separate set of 240 5-ns simulations (60 for each applied force) were performed with a given host-guest pair initially in the free (unbound) state. The correlation coefficient (value ranges from -1 to 1) between the direction of the applied force and the movement of the free guest molecule closest to the HA end being pulled was then determined. Values in the parentheses indicate the standard error of the mean from 60 simulations performed at a given force. The diffusion coefficient (D) of such movement was determined from a linear regression model fit to the mean square displacement of the corresponding guest molecule averaged over 60 simulations performed at a given force. Values in the parentheses indicate the standard error in the linear regression estimate of D.

**Force (pN)**	**System**	**Unbinding event**	**Correlation coefficient**	**D (nm^2/ns)**
10	HA-ADA	0/60	0.104 (0.023)	0.117 (0.001)
20	HA-ADA	0/60	0.291 (0.025)	0.165 (0.000)
50	HA-ADA	0/60	0.552 (0.033)	0.622 (0.006)
102	HA-ADA	2/60	0.702 (0.031)	1.844 (0.010)
10	HA-CA	-	0.159 (0.030)	0.125 (0.000)
20	HA-CA	-	0.260 (0.023)	0.135 (0.002)
50	HA-CA	-	0.597 (0.027)	0.545 (0.003)
102	HA-CA	0/60	0.787 (0.016)	1.389 (0.008)

Supplementary Table 4. Western blot analysis of pFAK, Runx 2 (after 3 days) and ALP (after 7 days) protein expression in hMSCs across various groups (HA-ADA-cRGD, HA-ADA-pRGD and HA-ADA) of osteogenic culture.

	HA-ADA-cRGD	HA-ADA-pRGD	HA-ADA
**Relative intensity of pFAK**	1.38	0.56	0.26
**Relative intensity of Runx 2**	1.00	0.91	0.58
**Relative intensity of ALP**	1.21	1.08	0.96

Supplementary Table 5. Thermodynamic binding parameters of HA-ADA and HA-CA with β-CD obtained from ITC measurements.

**System**	**ΔH (kcal mol^{-1})**	**TΔS (kcal mol^{-1})**	**K_{eq} (M^{-1})**
**HA-ADA**	-4.5	2.6	1.68×10^5
**HA-CA**	-2.7	3.9	7.13×10^4

Supplementary Table 6. Information of the primers used for real-time PCR. (Applied Biosystem)

Gene Name	Gene Expression Assay
**GAPDH**	**Hs02786624_g1**
**COL I**	**Hs00164004_m1**
**ALP**	**Hs01029144_m1**
**Runx 2**	**Hs00231692_m1**
**Vinculin**	**Hs01009014_m1**

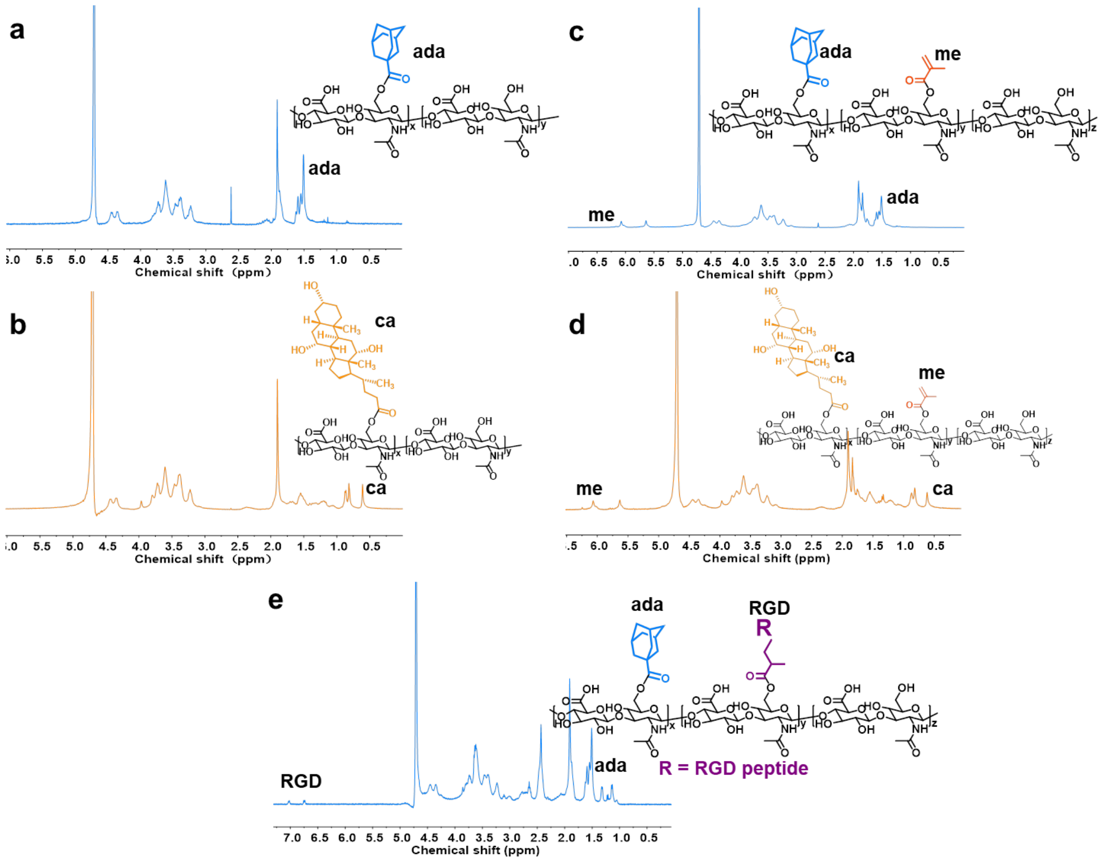

Supplementary Fig. 1 Characterization of synthesized macromers. ^{1}H NMR spectrum of **a** HA-ADA, **b** HA-CA, **c** methacrylated HA-ADA, **d** methacrylated HA-CA and **e** HA-ADA-cRGD.

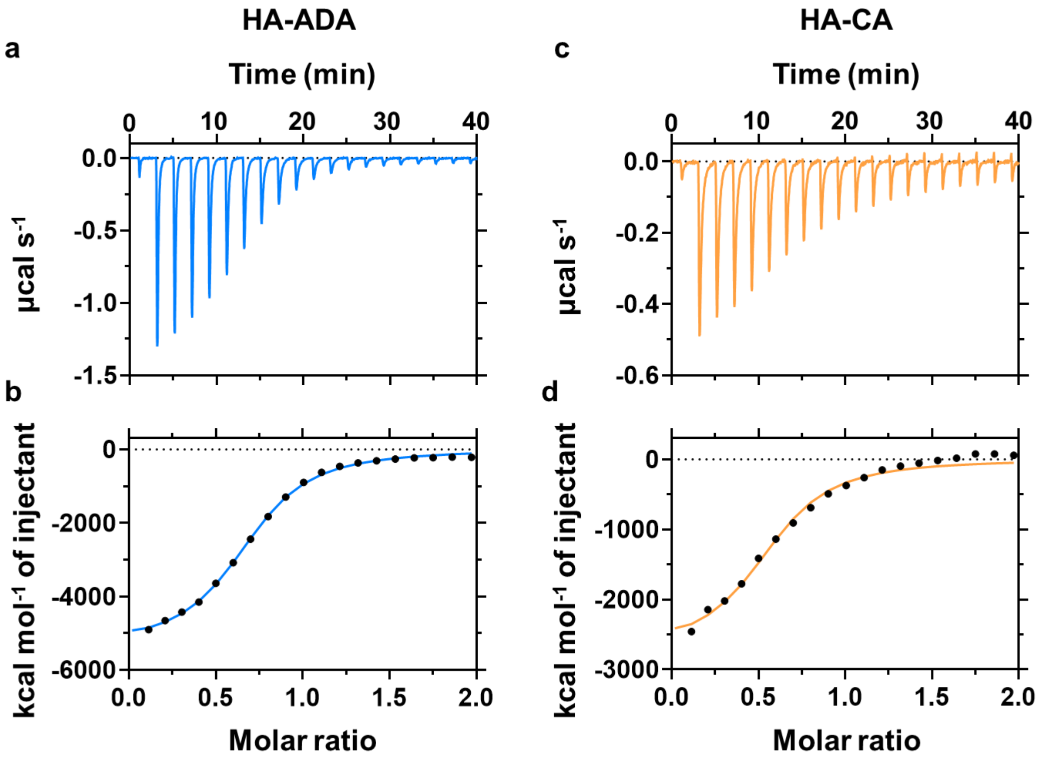

Supplementary Fig. 2 a Raw data for the titration of HA-ADA with ac-β-CD at pH 7.4, showing that the calorimetric response as successive injections of ac-CD is added to the sample cell. **b** Integrated heat profile of the calorimetric titration shown in panel a. **c** Raw data for the titration of HA-CA with ac-β-CD at pH 7.4, showing that the calorimetric response as successive injections of ac-β-CD is added to the sample cell. **d** Integrated heat profile of the calorimetric titration shown in panel c.

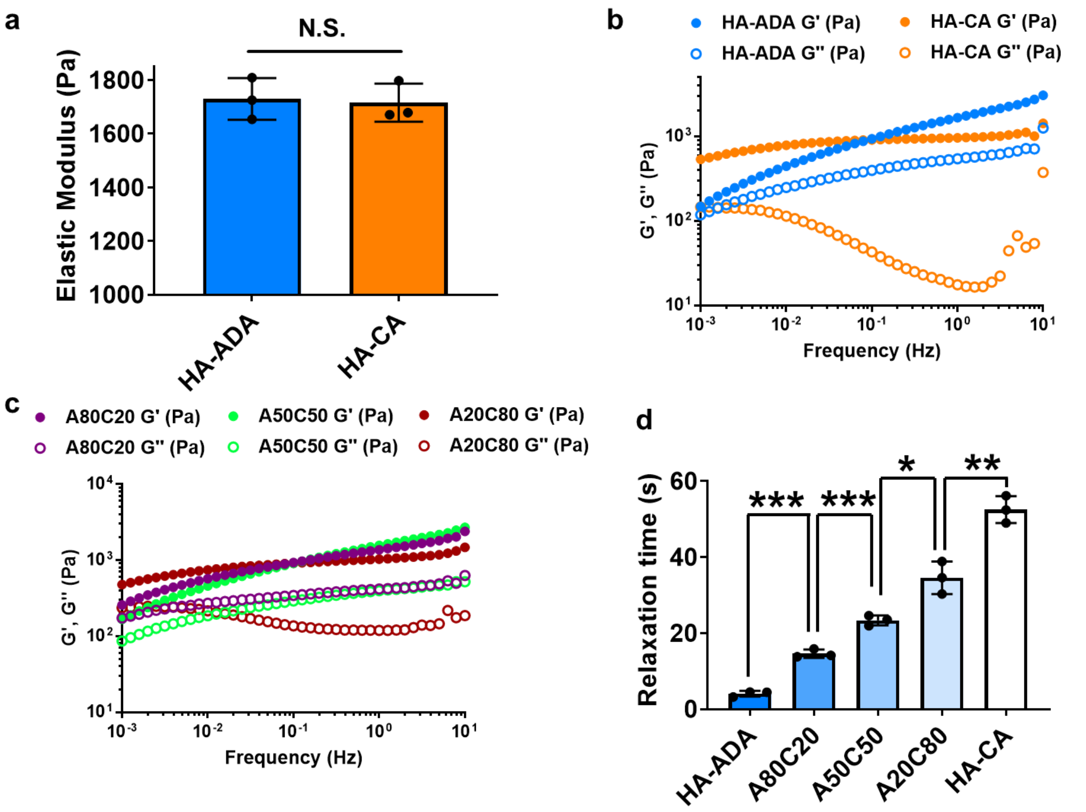

Supplementary Fig. 3 Mechanical characterization of the various hydrogels. a Elastic modulus of the HA-ADA and HA-CA hydrogels. **b** Shear storage (G') and shear loss (G") moduli of hydrogels (HA-ADA and HA-CA) during frequency sweep rheological analysis. **c** Shear storage (G') and shear loss (G") moduli of hydrogels (A80C20, A50C50 and A20C80) during frequency sweep rheological analysis. **d** Half-stress relaxation times for the various hydrogels investigated (Data are presented as mean values ± SD, n = 3 independent samples per group) *p < 0.05, **p < 0.01, ***p < 0.001, N.S. indicates no statistical difference (two-tailed Student's t-test). (A80C20, A50C50, A20C80 were prepared by mixing HA-ADA and HA-CA at the weight ratio of 80%:20%, 50%:50%, or 20%:80%, respectively)

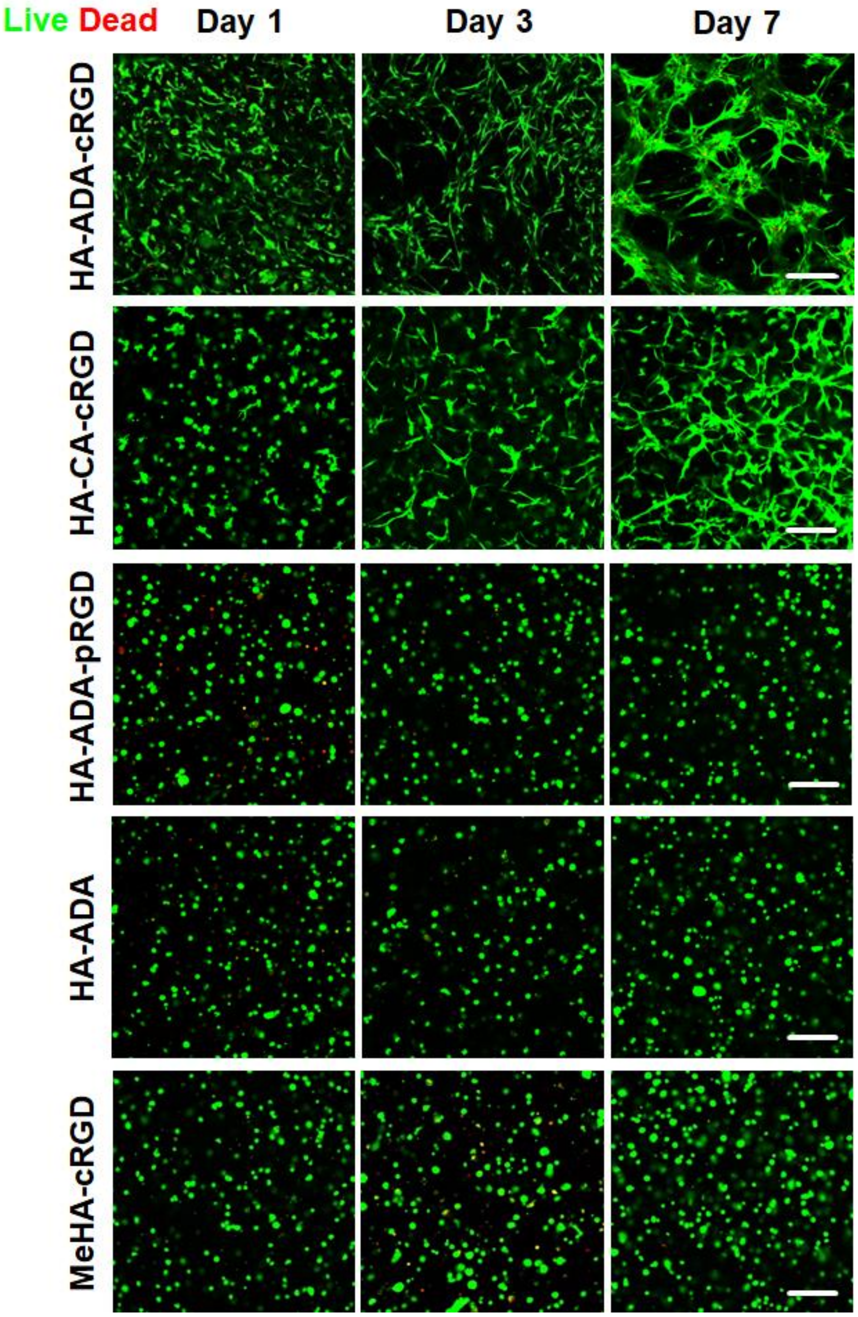

Supplementary Fig. 4 Encapsulated hMSC behaviour and viability. Representative live/dead staining images of hMSCs encapsulated in hydrogels with various RGD coupling methods after culture for 1, 3, and 7 days. Scale bar = 200 µm.

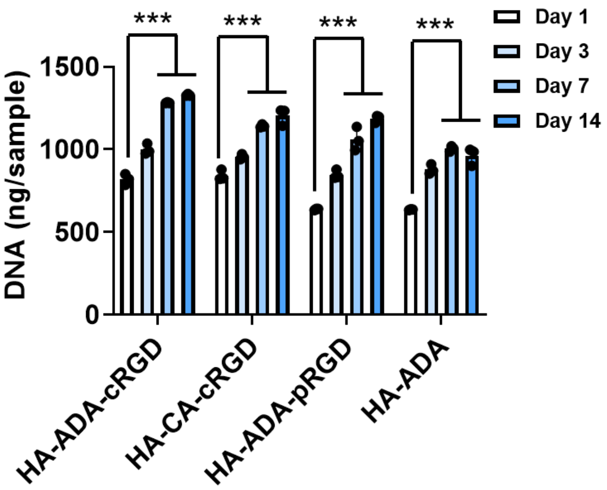

Supplementary Fig. 5 DNA content across various hydrogels. DNA isolated from hMSC-laden hydrogels with varying crosslinking methods and RGD coupling methods during 14 days of culture (Data are presented as mean values ± SD, $n = 3$ independent hydrogels) $***p < 0.001$ (two-tailed Student's t-test).

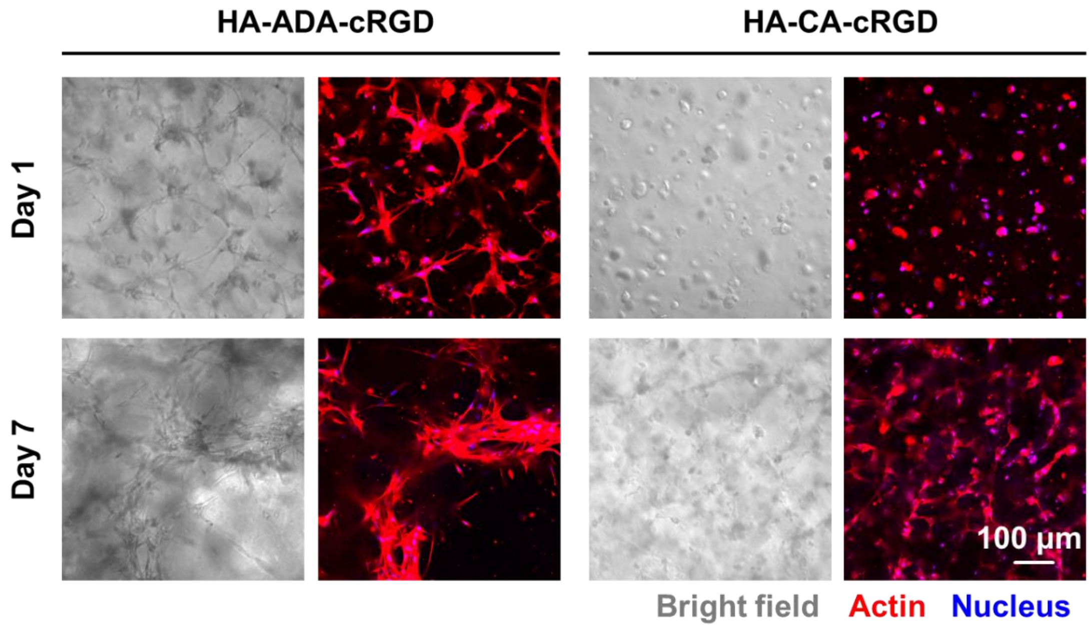

Supplementary Fig. 6 Encapsulated hMSC morphology. Representative optical micrographic image of hMSCs encapsulated in hydrogels from different groups (HA-ADA-cRGD and HA-CA-cRGD) on culture days 1 and 7, actin (red) and nuclei (blue). Scale bar =100 μm.

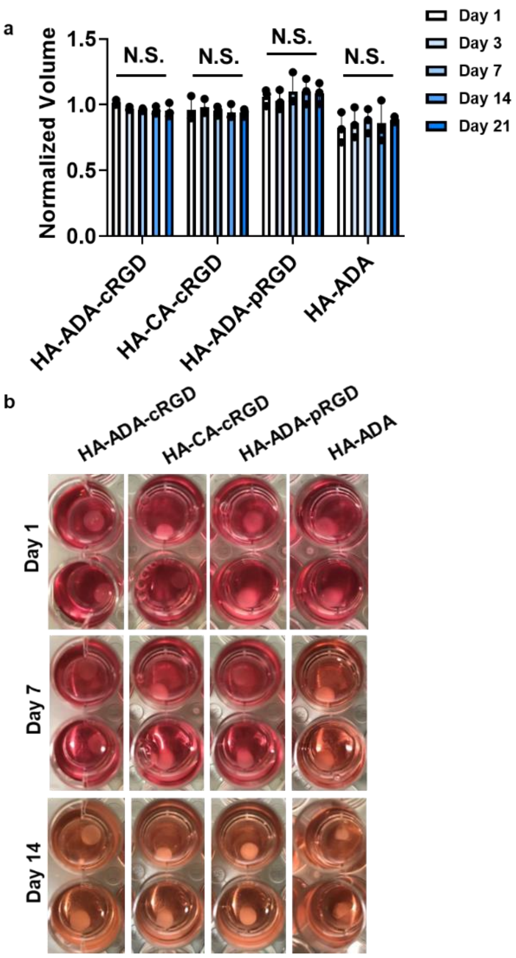

Supplementary Fig. 7 Hydrogel swelling with culture. a The normalized volume of cell-laden hydrogels with varying crosslink methods and RGD coupling methods measured after 1, 3, 7, 14 and 21 days of culture (Data are presented as mean values ± SD, n = 3 independent hydrogels) N.S. denotes no statistical differences (ANOVA). **b** Images of hydrogels with varying crosslink methods and RGD coupling methods after 1, 7 and 14 days of culture; the size of the hydrogel constructs was similar across the various groups.

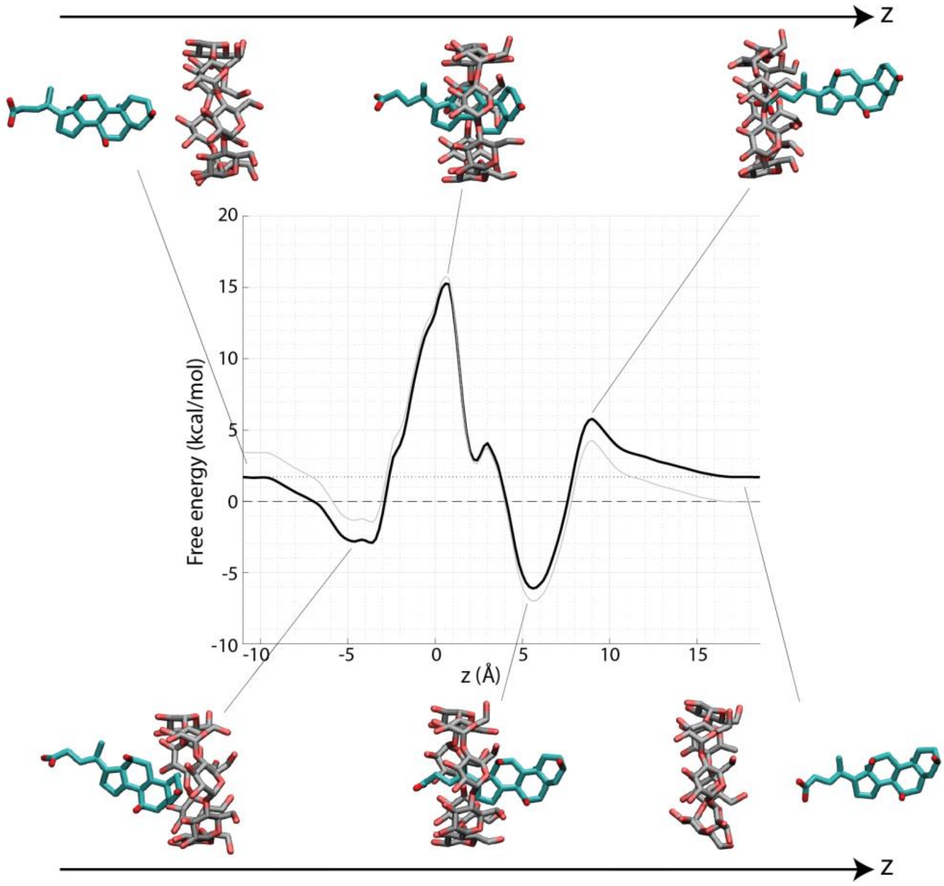

Supplementary Fig. 8 Potential of mean force (PMF) for CD-CA binding obtained from ABF calculations. Structures of the CD-CA complexation at six representative locations along the reaction coordinate (*z*) are highlighted. The thin gray curve represents the un-symmetrized PMF, while the thick black curve indicates the symmetrized result (see text for more details).

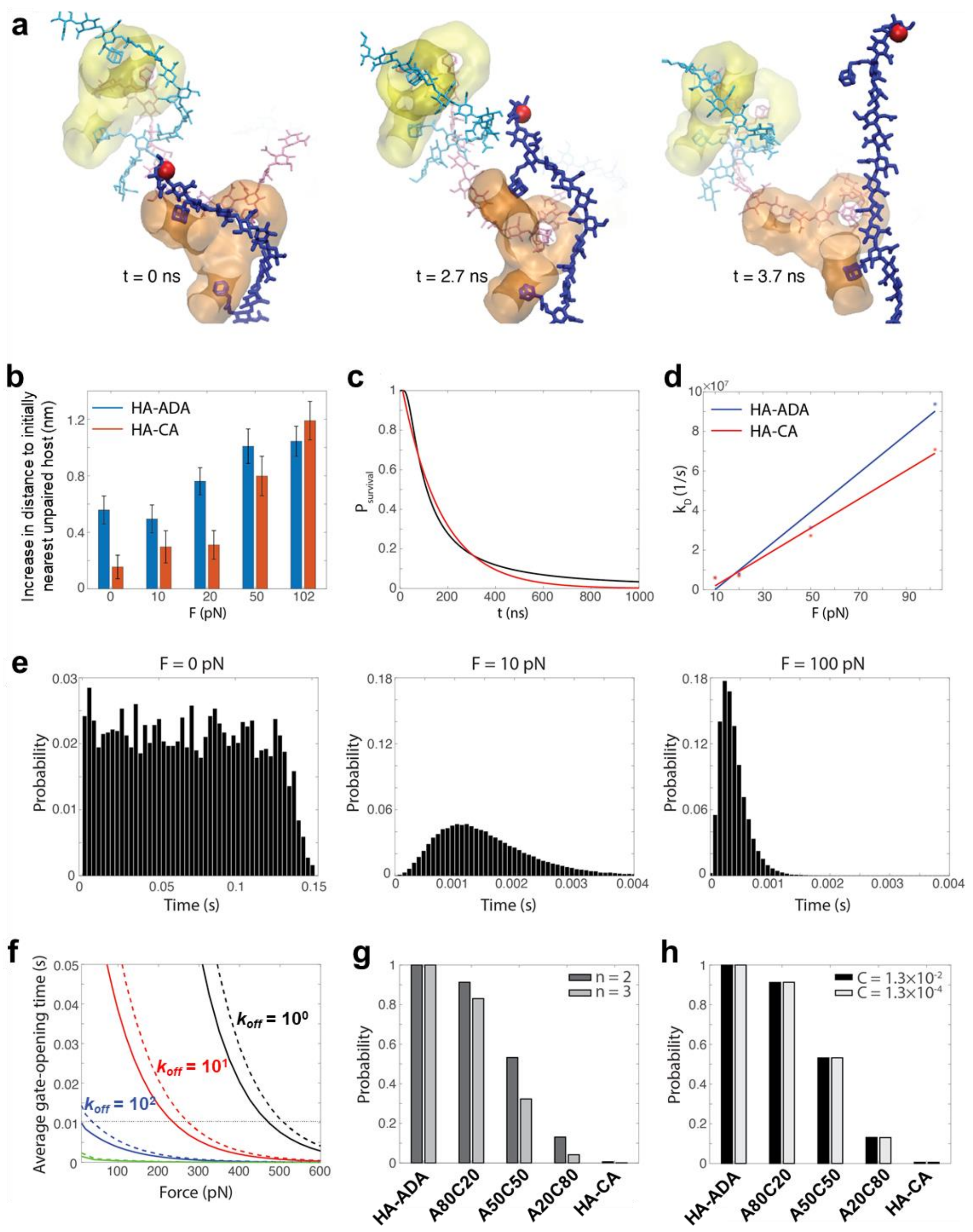

Supplementary Fig. 9 MD and KMC modelling. a MD simulation snapshots showing the unbinding process of a host-guest pair in HA-ADA under an external force of 102 pN applied tangentially along one end (red dot) of the HA segment. In altogether sixty simulations performed at F = 102 pN, unbinding of CD-ADA was recorded in two simulations. **b** The change in the distance between the free guest molecule (the one closest to the HA end being pulled) with its initially nearest unpaired host molecule increases with increasing magnitude of force applied, indicating a decreasing probability of reunion of the unbound host/guest molecules with their original partners. Error bars represent the standard error of the mean from 60 simulations performed at a given applied force. **c** $P_{survival}$ as a function of time (black) according to Equation (7) at F = 10 pN in HA-ADA. The fitted exponential form is shown in red, from which an effective rate constant k_D is obtained. **d** k_D obtained for F = 10, 20, 50 and 102 pN (dots)

and the linear fitting result (line) for HA-ADA (blue) and HA-CA (red), respectively. **e** Distribution of the gate-opening time in HA-ADA under a force of 0 pN, 10 pN or 100 pN, assuming that an actin filament faces four 2-host complexes. **f** Average gate-opening time with the thermodynamic constant K_{eq} kept unchanged, and k_{on} and k_{off} scaled down by 1-3 orders of magnitude from those of HA-ADA (green), assuming that an actin filament faces four 2-host complexes (solid) or four 3-host complexes (dashed), respectively. The blue curves correspond to k_{on} = 10^7 $M^{-1}s^{-1}$ and k_{off} = 10^2 s^{-1}, the red curves correspond to k_{on} = 10^6 $M^{-1}s^{-1}$ and k_{off} = 10^1 s^{-1}, and the black curves correspond to k_{on} = 10^5 $M^{-1}s^{-1}$ and k_{off} = 1 s^{-1}. The dotted lines indicate the timescale of actin polymerization (~0.01 s). **g** Probability for a gate-opening event to occur within the estimated timescale of actin polymerization (~0.01 s) at F = 100 pN, with n = 2 or n = 3 in acrylated host complexes. **h** The same probability shown in **g** with F = 100 pN and n = 2, where the k_{off} value of CA was varied from 1.3×10^{-2} s^{-1} to 1.3×10^{-4} s^{-1}.

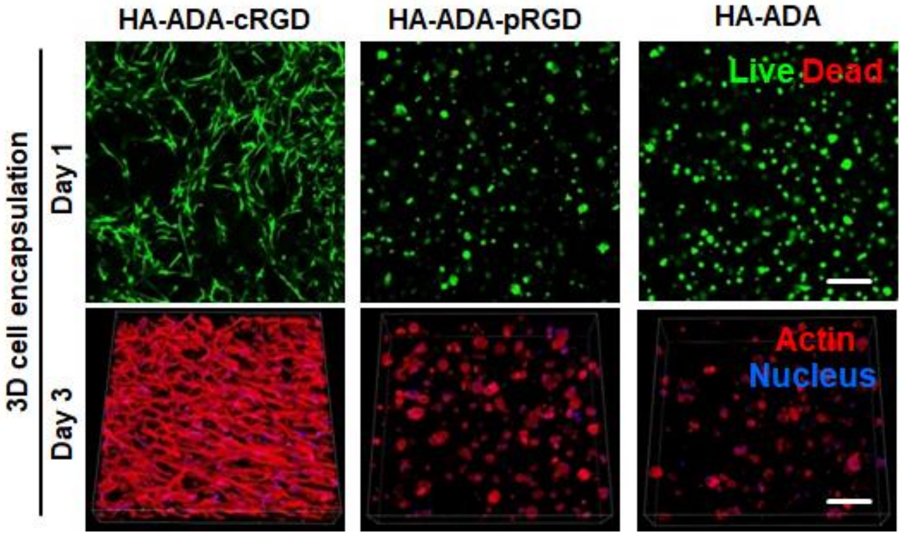

Supplementary Fig. 10 Representative images of hMSCs encapsulated within hydrogels (3D cell encapsulation) with different RGD conjugation methods. Representative images of hMSCs encapsulated within hydrogels (3D cell encapsulation) with different RGD conjugation methods (HA-ADA-cRGD, HA-ADA-pRGD and HA-ADA-without RGD) after 1 and 3 days of culture. Scale bar = 200 μm.

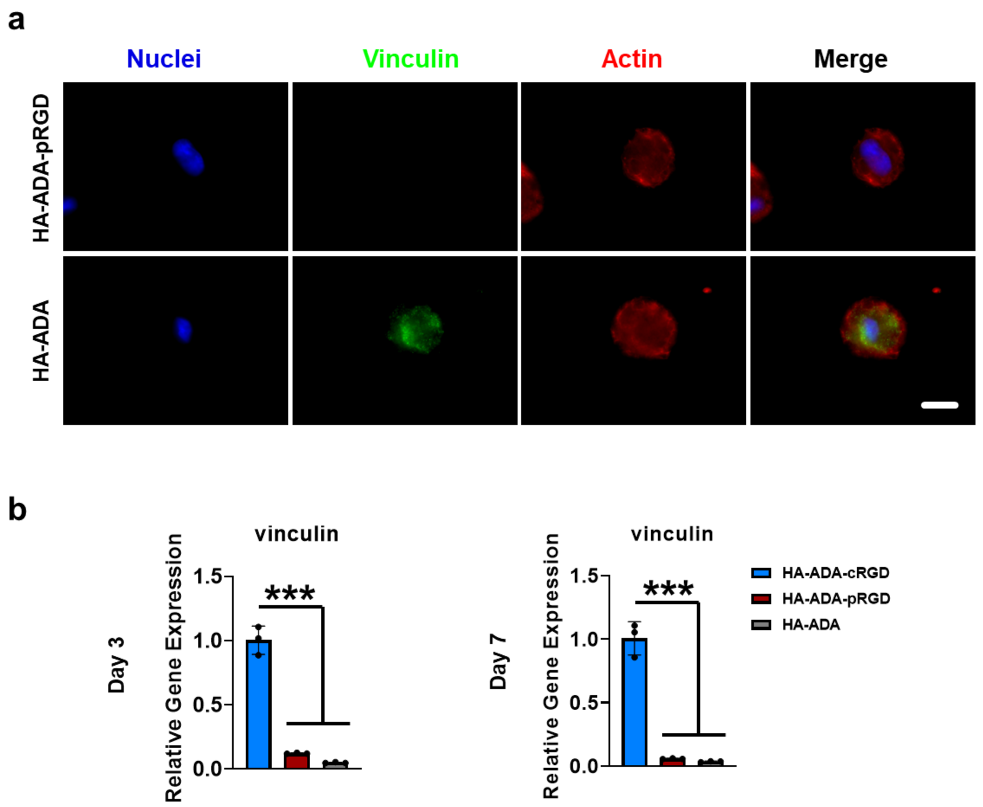

Supplementary Fig. 11 hMSC adhesion in 3D hydrogels. a Representative immunofluorescence staining for F-actin (red), nuclei (blue) and vinculin (green) in hMSCs cultured in the hydrogels (HA-ADA-pRGD and HA-ADA) for 3 days. Scale bar = 20 μm. **b** RT-PCR quantification of vinculin expression by hMSCs encapsulated in the hydrogels. Data are presented as mean values ± SD, $n = 3$ independent hydrogels; ***$p < 0.001$ (two-tailed Student's t-test).

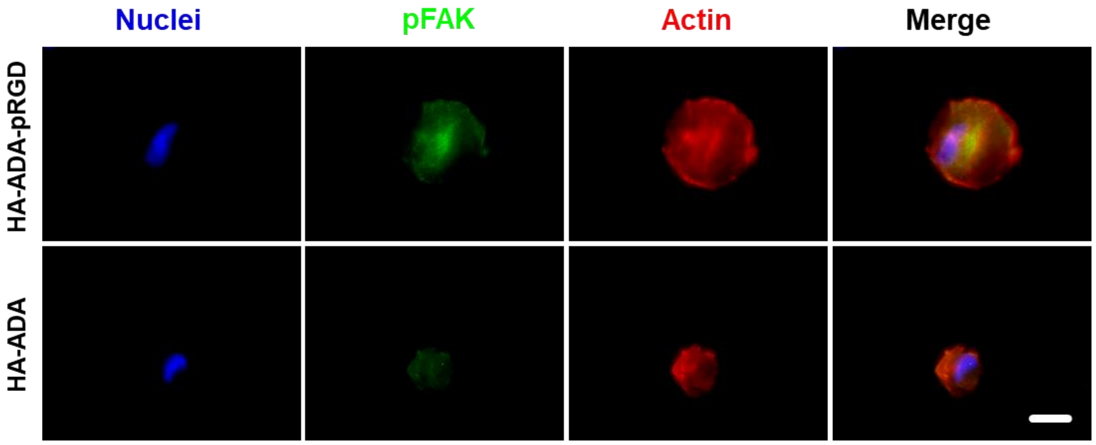

Supplementary Fig. 12 hMSC pFAK visualization in 3D hydrogels. Representative immunofluorescence staining for F-actin (red), nuclei (blue) and pFAK (green) in hMSCs cultured in the hydrogels (HA-ADA-pRGD and HA-ADA) for 3 days. Scale bar = 20 μm.

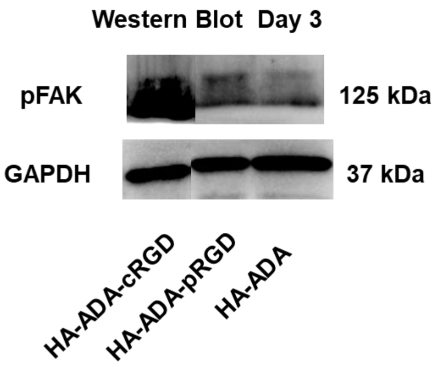

Supplementary Fig. 13 hMSC pFAK expression in 3D hydrogels. Western blot analysis of pFAK protein expression in hMSCs after 3 days of osteogenic culture in hydrogels prepared with different RGD coupling methods. The samples derive from the same experiment and that gels/blots were processed in parallel. (Data of Relative intensity details are in the Supplementary Table 4)

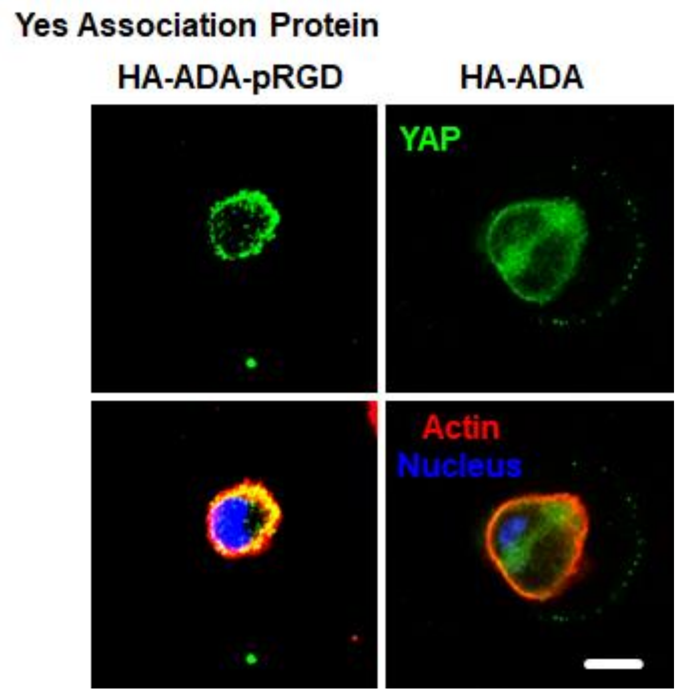

Supplementary Fig. 14 hMSC YAP staining in 3D hydrogels. Representative immunofluorescence staining for F-actin (red), nuclei (blue) and YAP (green) in hMSCs cultured in the hydrogels (HA-ADA-pRGD and HA-ADA) for 3 days. Scale bar = 20 μm.

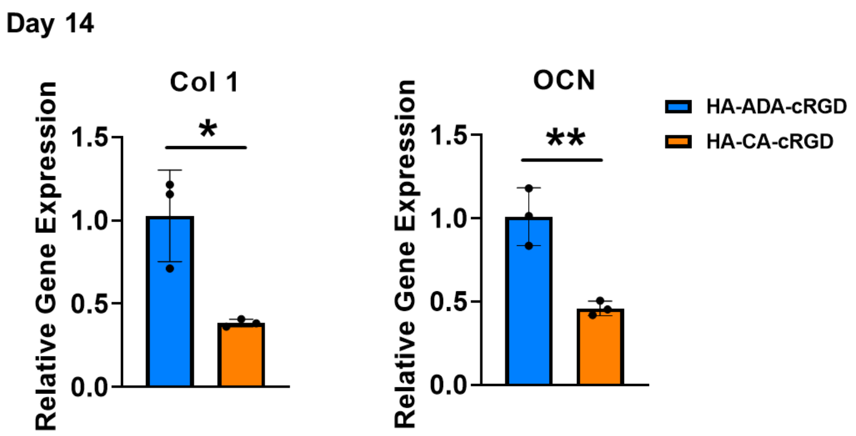

Supplementary Fig. 15 hMSC gene expression in 3D hydrogels. RT-PCR quantification of Col 1 and OCN expression by hMSCs encapsulated in the hydrogels (HA-ADA-cRGD and HA-CA-cRGD). Values are normalized to expression levels within HA-ADA. Data are presented as mean values ± SD, $n = 3$ independent hydrogels; *$p < 0.05$, **$p < 0.01$ (two-tailed Student's *t*-test).

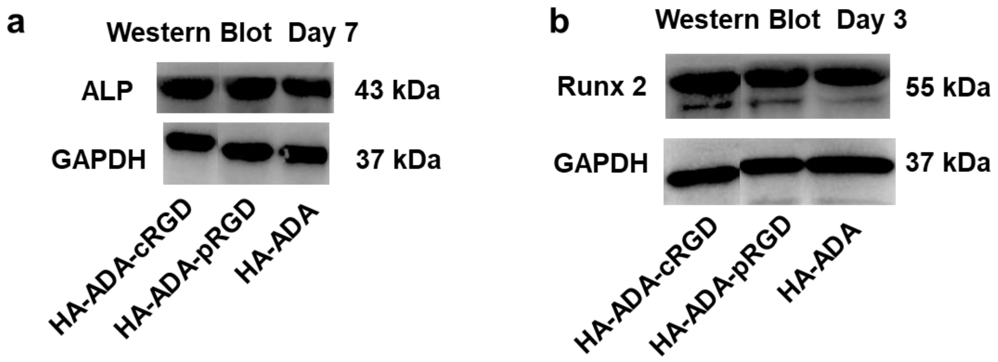

Supplementary Fig. 16 hMSC osteogenic protein expression in 3D hydrogels. Western blot analysis of **a** ALP (after 7 days) and **b** Runx 2 (after 3 days) protein expression in hMSCs across various groups (HA-ADA-cRGD, HA-ADA-pRGD and HA-ADA) of osteogenic culture. The samples derive from the same experiment and that gels/blots were processed in parallel. (Data of Relative intensity details are in the Supplementary Table 4)

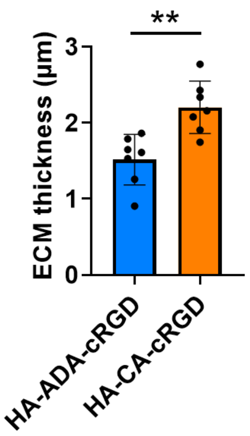

Supplementary Fig. 17 Nascent local ECM thickness around hMSCs in 3D hydrogels. Quantification of the average thickness of ECM deposited by hMSCs encapsulated in HA-ADA-cRGD and HA-CA-cRGD hydrogels after 18 hours of osteogenic culture. Data are presented as mean values ± SD, $n = 7$ cells per group from 2 independent experiments, $^{**}p < 0.01$ (two-tailed Student's t-test).

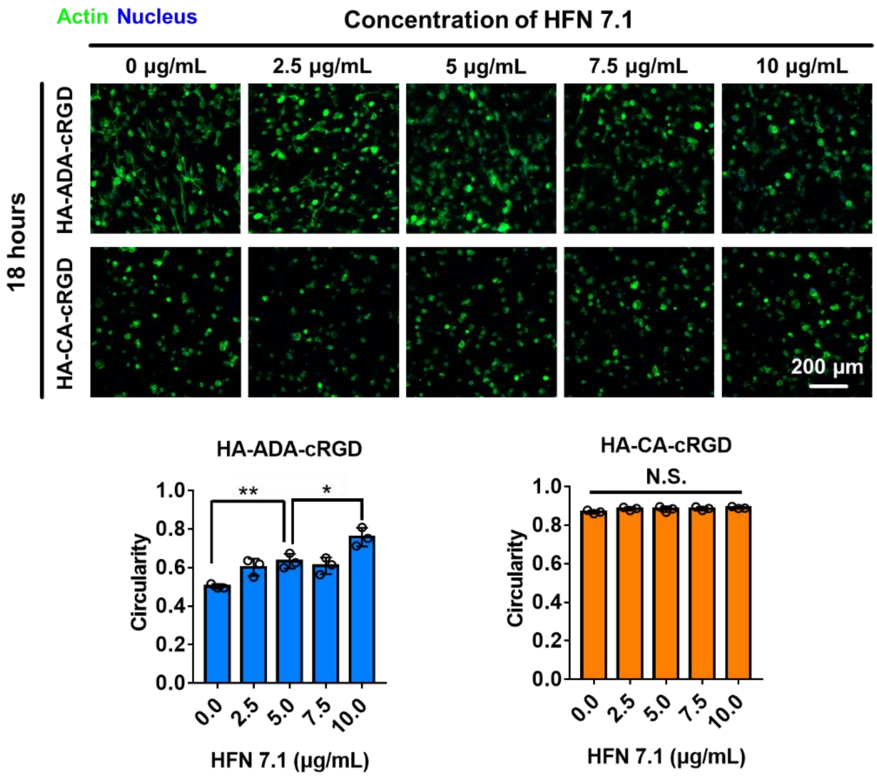

Supplementary Fig. 18 hMSC spreading with fibronectin blocking. Representative images of F-actin expressed by hMSCs encapsulated in HA-ADA-cRGD and HA-CA-cRGD hydrogels after 18 hours of treatment with different concentrations of monoclonal antibody against human fibronectin (HFN 7.1, 0, 5, 7.5, 10 $\mu g \cdot mL^{-1}$). Scale bars=200 µm. Circularity of hMSCs encapsulated in HA-ADA-cRGD and HA-CA-cRGD hydrogels after 18 hours with treatment of various concentrations of HFN 7.1 (0, 2,5, 5, 7.5, 10 $\mu g \cdot mL^{-1}$). Data are presented as mean values ± SD, n = 3 hydrogels; *p < 0.05, **p < 0.01, N.S. denotes no statistical significance (two-tailed Student's t-test or ANOVA).

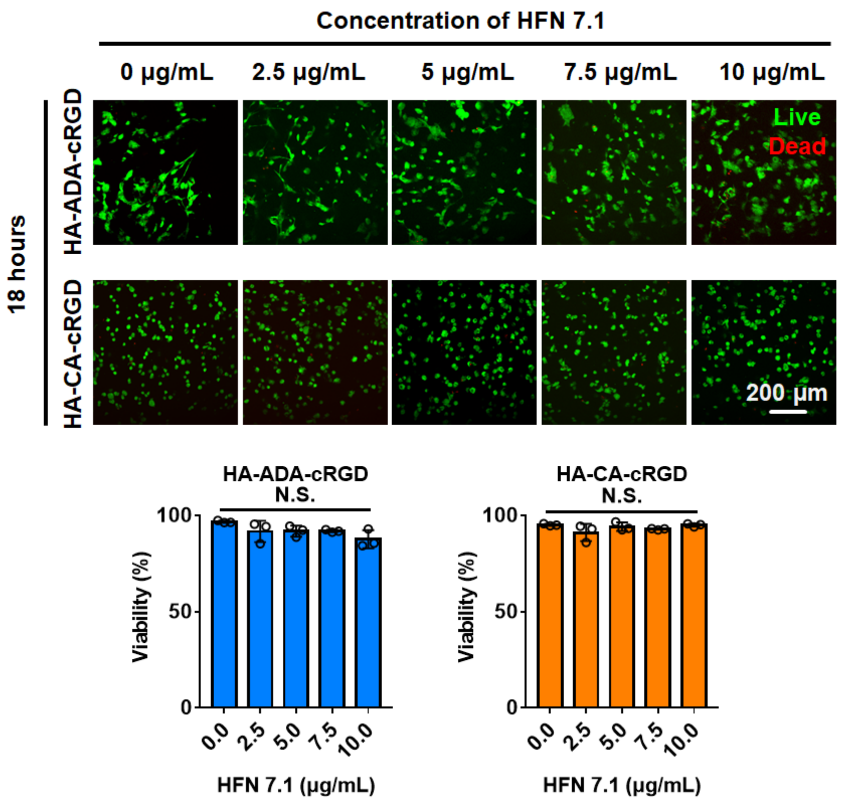

Supplementary Fig. 19 hMSC viability during fibronectin blocking studies. Viability of hMSCs encapsulated in HA-ADA-cRGD and HA-CA-cRGD hydrogels with treatment of various concentrations of monoclonal antibody against human fibronectin (HFN 7.1, 0, 2.5, 5, 7.5, 10 μg·mL^{-1}). Scale bar = 200 μm. Quantification of the viability of hMSCs encapsulated in HA-ADA-cRGD and HA-CA-cRGD hydrogels with treatment of various concentrations of HFN 7.1 (0, 2.5, 5, 7.5, 10 μg·mL^{-1}). Data are presented as mean values ± SD, n = 3 hydrogels per group; N.S. denotes no statistical differences (ANOVA).

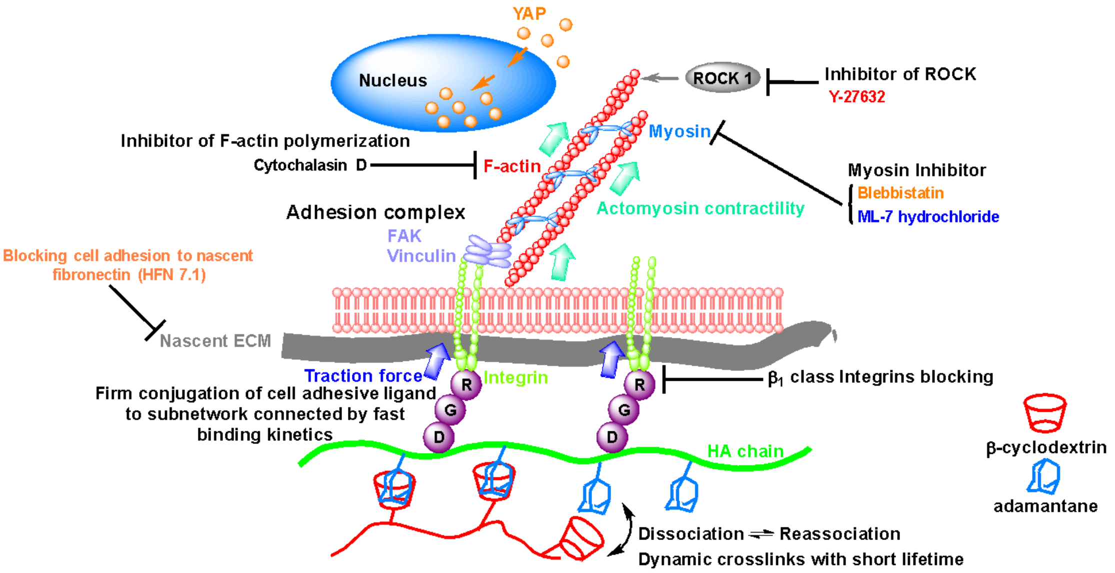

Supplementary Fig. 20. Schematic illustration of the molecular mechanisms underlying the ultra-fast spreading and mechanosensing of hMSCs in the HA-ADA-cRGD hydrogel.

References

1. Feng, Q. et al. Mechanically resilient, injectable, and bioadhesive supramolecular gelatin hydrogels crosslinked by weak host-guest interactions assist cell infiltration and in situ tissue regeneration. *Biomaterials* **101**, 217-228 (2016).
2. Wei, K.C. et al. Robust Biopolymeric Supramolecular "Host-Guest Macromer" Hydrogels Reinforced by in Situ Formed Multivalent Nanoclusters for Cartilage Regeneration. *Macromolecules* **49**, 866-875 (2016).
3. Rodell, C.B., Kaminski, A.L. & Burdick, J.A. Rational Design of Network Properties in Guest-Host Assembled and Shear-Thinning Hyaluronic Acid Hydrogels. *Biomacromolecules* **14**, 4125-4134 (2013).
4. Rosales, A.M., Vega, S.L., DelRio, F.W., Burdick, J.A. & Anseth, K.S. Hydrogels with Reversible Mechanics to Probe Dynamic Cell Microenvironments. *Angew Chem Int Edit* **56**, 12132-12136 (2017).
5. Bell, G.I. Theoretical-Models for the Specific Adhesion of Cells to Cells or to Surfaces. *Adv Appl Probab* **12**, 566-567 (1980).
6. Chan, C.E. & Odde, D.J. Traction Dynamics of Filopodia on Compliant Substrates. *Science* **322**, 1687-1691 (2008).
7. Auletta, T. et al. beta-cyclodextrin host-guest complexes probed under thermodynamic equilibrium: Thermodynamics and AFM force spectroscopy. *J Am Chem Soc* **126**, 1577-1584 (2004).
8. Darve, E., Rodriguez-Gomez, D. & Pohorille, A. Adaptive biasing force method for scalar and vector free energy calculations. *J Chem Phys* **128** (2008).
9. Henin, J., Fiorin, G., Chipot, C. & Klein, M.L. Exploring Multidimensional Free Energy Landscapes Using Time-Dependent Biases on Collective Variables. *J Chem Theory Comput* **6**, 35-47 (2010).
10. Cabrer, P.R. et al. Complexation of sodium cholate and sodium deoxycholate by beta-cyclodextrin and derivatives. *Langmuir* **15**, 5489-5495 (1999).
11. Schonbeck, C. Complexation Kinetics of Cyclodextrins with Bile Salt Anions: Energy Barriers for the Threading of Ionic Groups. *J Phys Chem B* **123**, 9831-9838 (2019).
12. Guvench, O., Hatcher, E., Venable, R.M., Pastor, R.W. & MacKerell, A.D. CHARMM Additive All-Atom Force Field for Glycosidic Linkages between Hexopyranoses. *J Chem Theory Comput* **5**, 2353-2370 (2009).
13. Guvench, O. et al. Additive Empirical Force Field for Hexopyranose Monosaccharides. *J Comput Chem* **29**, 2543-2564 (2008).
14. Vanommeslaeghe, K. et al. CHARMM General Force Field: A Force Field for Drug-Like Molecules Compatible with the CHARMM All-Atom Additive Biological Force Fields. *J Comput Chem* **31**, 671-690 (2010).
15. Vanommeslaeghe, K. & MacKerell, A.D. Automation of the CHARMM General Force Field (CGenFF) I: Bond Perception and Atom Typing. *J Chem Inf Model* **52**, 3144-3154 (2012).
16. Vanommeslaeghe, K., Raman, E.P. & MacKerell, A.D. Automation of the CHARMM General Force Field (CGenFF) II: Assignment of Bonded Parameters and Partial Atomic Charges. *J Chem Inf Model* **52**, 3155-3168 (2012).
17. Phillips, J.C. et al. Scalable molecular dynamics with NAMD. *J Comput Chem* **26**, 1781-1802 (2005).
18. Andersen, H.C. Rattle - a Velocity Version of the Shake Algorithm for Molecular-Dynamics Calculations. *J Comput Phys* **52**, 24-34 (1983).

19. Miyamoto, S. & Kollman, P.A. Settle - an Analytical Version of the Shake and Rattle Algorithm for Rigid Water Models. *J Comput Chem* **13**, 952-962 (1992).
20. Darden, T., York, D. & Pedersen, L. Particle Mesh Ewald - an N.Log(N) Method for Ewald Sums in Large Systems. *J Chem Phys* **98**, 10089-10092 (1993).
21. Feller, S.E., Zhang, Y.H., Pastor, R.W. & Brooks, B.R. Constant-Pressure Molecular-Dynamics Simulation - the Langevin Piston Method. *J Chem Phys* **103**, 4613-4621 (1995).
22. Gumbart, J.C., Roux, B. & Chipot, C. Standard Binding Free Energies from Computer Simulations: What Is the Best Strategy? *J Chem Theory Comput* **9**, 794-802 (2013).
23. Steketee, M.B. & Tosney, K.W. Three functionally distinct adhesions in filopodia: Shaft adhesions control lamellar extension. *J Neurosci* **22**, 8071-8083 (2002).
24. Lewis, A.K. & Bridgman, P.C. Nerve Growth Cone Lamellipodia Contain 2 Populations of Actin-Filaments That Differ in Organization and Polarity. *J Cell Biol* **119**, 1219-1243 (1992).
25. Svitkina, T.M. et al. Mechanism of filopodia initiation by reorganization of a dendritic network. *J Cell Biol* **160**, 409-421 (2003).
26. Ishikawa, R., Sakamoto, T., Ando, T., Higashi-Fujime, S. & Kohama, K. Polarized actin bundles formed by human fascin-1: their sliding and disassembly on myosin II and myosin V in vitro. *J Neurochem* **87**, 676-685 (2003).
27. Claessens, M.M.A.E., Semmrich, C., Ramos, L. & Bausch, A.R. Helical twist controls the thickness of F-actin bundles. *P Natl Acad Sci USA* **105**, 8819-8822 (2008).
28. Oda, T., Iwasa, M., Aihara, T., Maeda, Y. & Narita, A. The nature of the globular-to fibrous-actin transition. *Nature* **457**, 441-445 (2009).
29. Takagi, J., Petre, B.M., Walz, T. & Springer, T.A. Global conformational rearrangements in integrin extracellular domains in outside-in and inside-out signaling. *Cell* **110**, 599-611 (2002).
30. Coller, B.S. & Shattil, S.J. The GPIIb/IIIa (integrin alpha IIb beta 3) odyssey: a technology-driven saga of a receptor with twists, turns, and even a bend. *Blood* **112**, 3011-3025 (2008).
31. Galior, K., Liu, Y., Yehl, K., Vivek, S. & Salaita, K. Titin-Based Nanoparticle Tension Sensors Map High-Magnitude Integrin Forces within Focal Adhesions. *Nano Lett* **16**, 341-348 (2016).
32. Wang, X.F. & Ha, T. Defining Single Molecular Forces Required to Activate Integrin and Notch Signaling. *Science* **340**, 991-994 (2013).
33. Sabass, B., Gardel, M.L., Waterman, C.M. & Schwarz, U.S. High resolution traction force microscopy based on experimental and computational advances. *Biophys J* **94**, 207-220 (2008).
34. Chang, A.C. et al. Single Molecule Force Measurements in Living Cells Reveal a Minimally Tensioned Integrin State. *Acs Nano* **10**, 10745-10752 (2016).
35. Yim, C.T., Zhu, X.X. & Brown, G.R. Kinetics of inclusion reactions of beta-cyclodextrin with several dihydroxycholate ions studied by NMR spectroscopy. *J Phys Chem B* **103**, 597-602 (1999).
36. Voter, A.F. Introduction to the Kinetic Monte Carlo Method. *Nato Sci Ser Ii-Math* **235**, 1-23 (2007).
37. Nelson, P. Biological physics. (WH Freeman New York, 2004).
