# Supplementary material for: Enhanced mechanosensing of cells in synthetic 3D matrix with controlled biophysical dynamics
Source: Nat Commun. 2021 Jun 10;12:3514. doi: 10.1038/s41467-021-23120-0 (PMC8192531; doi:10.1038/s41467-021-23120-0)
Supplement: Supplementary file 3 — Description of Additional Supplementary Files [file 41467_2021_23120_MOESM3_ESM.docx]

**Description of Additional Supplementary Files**

**Supplementary Movie 1.** Cells encapsulated in the HA-ADAcRGD hydrogel 0-24 h

**Supplementary Movie 2.** Cells encapsulated in the HA-ADAcRGD hydrogel day 3 (3D reconstruction of hMSCs from confocal imaging)

**Supplementary Movie 3.** Cells encapsulated in the HA-ADApRGD hydrogel 0-24 h

**Supplementary Movie 4.** Cells encapsulated in the HA-ADApRGD hydrogel day 3
